# Laryngopharyngeal Reflux in Sleep-Disordered Breathing Patients

**DOI:** 10.22038/IJORL.2022.57515.2991

**Published:** 2022-11

**Authors:** Elvie Zulka Kautzia Rachmawati, Retno S. Wardhani, Rahmanofa Yunizaf, Saptawati Bardosono, Fikri Mirza Putranto, Niken Ageng Rizki, Nabilla Calista, Susyana Tamin

**Affiliations:** 1 *Department of Otorhinolaryngology Head and Neck Surgery, Faculty of Medicine Universitas Indonesia – Cipto Mangunkusumo Hopsital, Jakarta, Indonesia.*; 2 *Department Nutrition, Faculty of Medicine Universitas Indonesia – Cipto Mangunkusumo Hospital, Jakarta, Indonesia.*; 3 *Department of Otorhinolaryngology Head and Neck Surgery, Faculty of Medicine Universitas Indonesia – Universitas Indonesia Hospital, Depok, Indonesia.*

**Keywords:** Apnea-Hypopnea Index, Laryngopharyngeal Reflux, Nasolaryngopharyngeal Endoscopy, Obstructive Sleep Apnea, Reflux Symptom Index.

## Abstract

**Introduction:**

Our study aims to evaluate the distribution of laryngopharyngeal reflux (LPR) in patients with sleep-disordered breathing (SDB) via the Reflux Symptom Index (RSI) and to describe the sleep architecture in SDB patients with and without LPR.

**Materials and Methods:**

A cross-sectional, descriptive study was conducted. Patients with SDB were identified via the Epworth Sleepiness Scale (ESS) and STOP-BANG questionnaire; they were then screened with the RSI and physical examination for LPR. PSG was performed to evaluate obstructive sleep apnea (OSA).

**Results:**

Of 45 patients, 15 were scored as having LPR via the RSI. Utilizing the Respiratory Disturbance Index (RDI), patients were further classified into four groups: 9 non-LPR with non-OSA SDB, 21 non-LPR with OSA, 4 LPR with non-OSA SDB, and 11 LPR with OSA. The prevalence of LPR was 30.8% in the non-OSA SDB group and 34.4% in the OSA group. All SDB parameters in both groups were similar. SDB patients with high body mass index tended to have LPR and/or OSA. Average ESS scores in the four groups suggested excessive daytime sleepiness, and patients with LPR had higher ESS scores. Regardless of LPR status, SDB patients had a lower percentage of REM sleep and a higher percentage of light sleep.

**Conclusions:**

The incidence of LPR in OSA patients was similar in non-OSA SDB patients. REM sleep percentage decreased in the four groups, with the non-OSA SDB group having the lowest percentage of REM sleep; light sleep percentage increased in the four groups, with the OSA group having the highest percentage of light sleep.

## Introduction

Sleep-disordered breathing (SDB) is a spectrum of diseases characterized by abnormalities in respiration during sleep. The most common and severe form of SDB is obstructive sleep apnea (OSA); in this study, SDB was classified as non-OSA or OSA SDB. Diseases included in non-OSA SDB are primary snoring, central apnea, sleep-related hypoventilation and hypoxemia disorder, OSA hypopnea syndrome (OSAHS), and upper-airway resistance syndrome (UARS) (1,2).

OSA is a common syndrome characterized by recurring events of airway obstruction during sleep (3). OSA causes excessive daytime sleepiness (EDS), resulting in an increase in motor vehicle accidents, impaired cognitive function, and increased effort in breathing (4). The main risk factors for OSA are obesity, advanced age, and comorbidities, such as cardiovascular and metabolic diseases. Clinical evaluation for OSA is conducted using brief screening questionnaires like the Epworth Sleepiness Scale (ESS), STOP-BANG, and Berlin Questionnaire, followed by a polysomnogram sleep study (PSG) as the gold standard (5).

A laryngopharyngeal reflux (LPR) episode is defined as retrograde flow of stomach acid into the larynx and pharynx. The acid comes in contact with the mucosa of the aerodigestive tracts (especially hypopharynx and larynx), causing epithelial damage, inflammation, ciliary dysfunction, and altered sensitivity (6). The prevalence of LPR is 20–40% in the adult population (7). LPR is associated with vocal cord dysfunction, chronic obstructive pulmonary disease, and laryngeal cancer, which increase direct and indirect medical costs and decrease the quality of life of patients (7,8).

Several studies have reported an association between OSA and LPR; they share the same risk factors, such as old age and obesity, which may justify apnea and reflux in the same individual (3,9). The prevalence of OSA is 2–4% in adults (7). In a study conducted by Xavier et al. (10), the prevalence of signs and symptoms suggestive of LPR in adults with OSA was 89% higher in obese patients than non-obese patients. The relationship between OSA and LPR may be bidirectional. Several theories support this hypothesis, including the gradient pressure changes and inflammatory response theory. Gradient pressure changes in the abdomen and thorax during airway obstruction lead to relaxation of the lower esophageal sphincter (LES) and cause LPR. In addition, the inflammatory response in the pharynx due to refluxate causes delayed response of the pharyngeal dilator muscle and increases OSA severity (11,12).

However, the exact correlation of the underlying pathophysiology between OSA and LPR has not yet been established. A study by Erdem et al.(13) evaluated LPR distribution in OSA patients using a triple-sensor pH catheter and found that 83.9% of OSA patients could be diagnosed with LPR using the proximal probe. Another study by Iannella et al.(14) evaluated LPR in 75 OSA patients using the salivary pepsin concentration test and reported 32% positivity. We conducted this study to observe the distribution of LPR via the Reflux Symptom Index (RSI) and SDB and describe the sleep architecture in SDB with and without LPR (3).

## Materials and Methods

***Study Population and Study Design***

A cross-sectional, descriptive study using secondary retrospective data was performed at the Broncho-Esophagology Division of ORL-HNS Department, Cipto Mangunkusumo National Referral Hospital, to evaluate the distribution of LPR in OSA and describe the sleep architecture in SDB with and without LPR. Data were obtained from the medical records from January 2017 to April 2019. The study was been approved by the ethical committee of the Faculty of Medicine Universitas Indonesia (ethical number: 0840/UN2/F1/ETIK/2018) in August 2018. Using the sample size calculation in cross-sectional studies, we calculated the adequate sample size in the prevalence study, which was 40 subjects. This study included 45 subjects. The inclusion criteria of this study were patients over 18 years of age with complete medical records who had chief complaints of snoring or excessive day time sleepiness or witnessed apnea. All subjects required ESS, STOP-BANG, and RSI questionnaire, body mass index (BMI), and PSG data. The exclusion criteria included incomplete medical records and poor PSG data. The subjects were divided into two groups based on age, <55 years and ≥55 years, as sleep apnea is both and an age-related and age-dependent condition that peaks at the age of 55 years and slightly decreases afterward. The ESS is a questionnaire that evaluates EDS due to sleep disturbances. 

A score of more than 10/24 points suggests EDS. STOP-BANG is questionnaire with eight yes or no questions that determines the risk of having OSA; a score of 0–2 indicates low risk, 3–4 intermediate risk, and >5 high risk. Physical examinations were performed to evaluate the weight and height of patients whose BMI was >25 kg/m^2^ and defined as obese based on Asia-Pacific BMI Classification (15). The RSI is a 9-point questionnaire that assesses symptoms ranging from 0 (no problem) to 5 (severe problem). Patients with RSI >13 comprised the LPR group and patients with RSI ≤13 comprised the non-LPR group. PSG was performed using RESMED SOMNO touch RESP (level 2). The Respiratory Disturbance Index (RDI) was evaluated as a summation of Apnea Hypopnea Index (AHI) and respiratory effort-related arousal (RERA); a score of 5–15 suggested mild OSA, >15-30 moderate OSA, and >30 severe OSA. Minimum O_2_ saturation and percentage of REM, deep, and light sleep were also recorded. Statistical analysis was performed using SPSS 22.0 (IBM Corp., Armonk, NY, USA). Data were compared using the Chi-square test or Fisher’s exact test when one or more cell counts in the 2×2 table was less than 5. p <0.05 was considered statistically significant.

## Results

The RSI parameters were analyzed. RSI was assessed in 45 subjects: 30 were grouped as non-LPR and 15 as LPR. The non-LPR group included 13 male and 17 female subjects, and the LPR group included 10 male and 5 female subjects. 

Regarding SDB status, there were 9 non-OSA non-LPR SDB subjects, 21 OSA non-LPR subjects, 4 non-OSA LPR subjects, and 11 OSA LPR subjects. 

**Table 1:** Characteristics of non-LPR and LPR patients evaluated via RSI in accordance with OSA parameters

**Parameters (median)**	**Non-LPR (n = 30)**	**LPR (n = 15)**	***p-*** **value**
Age (years)			
Non-OSA SDB	44.00 (18.00-73.00)	48.00 (45.00-71.00)	0.503
OSA	44.00 (22.00-68.00)	52.00 (32.00-64.00)	0.667
BMI (kg/m^2^)			
Non-OSA SDB	24.00 (16.00-34.00)	24.50 (20.00-26.00)	1.00
OSA	27.00 (21.00-34.00)	25.00 (20.00-34.00)	0.584
ESS	10.77 ± 0.75	13.67 ± 1.56	0.064
STOPBANG	4.00 (2.00-8.00)	5.00 (3.00-8.00)	0.204
RDI (events/h)			
Non-OSA SDB	1.95 (0.30-4.00)	2.30 (2.10-3.20)	0.503
OSA	15.40 (5.40-35.30)	9.40 (5.10-75.90)	0.506
AHI (events/h)			
Non OSA SDB	1.10 (0.00-3.70)	0.80 (0.30-2.30)	0.503
OSA	13.60 (0.60-28.80)	5.30 (1.70-75.60)	0.755
RERA (events/h)			
Non-OSA SDB	0.70 (0.00-1.60)	1.80 (0.00-2.40)	0.940
OSA	3.20 (0.00-17.70)	2.00 (0.00-10.40)	0.367
Min. Sat O2 (%)	90.00 (66.00-95.00)	89.00 (44.00-93.00)	0.241
REM (%)			
Non-OSA SDB	2.40 (0.00-7.40)	3.10 (0.00-5.60)	0.414
OSA	5.90 (0.00-19.40)	5.20 (1.30-9.80)	0.969
Deep Sleep (%)			
Non-OSA SDB	24.40 (4.70-66.60)	32.60 (17.80-37.70)	1
OSA	18.20 (0.00-44.80)	20.10 (0.00-46.50)	0.785
Light Sleep (%)			
Non-OSA SDB	64.80 (30.60-95.30)	67.40 (56.70-79.10)	1
OSA	72.90 (51.30-97.30)	74.50 (48.10-95.30)	0.938

Out of 32 patients with OSA, 11 (34%) were diagnosed with LPR. 

The incidence of LPR in the non-OSA SDB group was 30.8%. 

Table 1 shows the classification of non-LPR and LPR subjects based on RSI. The two groups were not statistically different in almost all OSA parameters evaluated. 

**Fig 1:**
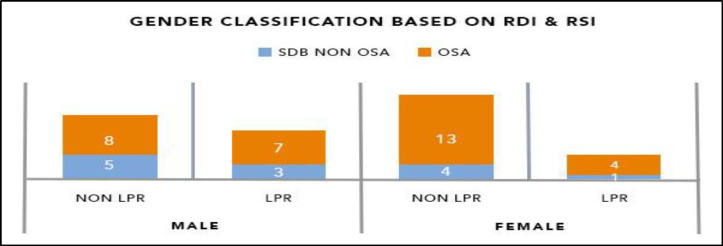
Gender classification based on RDI and RSI

Regarding gender, LPR was more likely to occur in males with SDB than in females (Figure 1). 

In non-OSA SDB subjects, the prevalence of LPR in males (38%) was almost twice the prevalence in females (20%). In the OSA group, 7/15 (46%) males with OSA had LPR; the prevalence in females was 24%. 

However, no statistically significant association between gender and occurrence of LPR was found in the non-OSA SDB (p=0.530) and OSA patients (p=0.450).

**Table 2:** BMI and Age Classification Based on RDI & RSI

		**LPR** **n (%)**	**Non-LPR** **n (%)**	**Total** **n (%)**	***p-*** value*
**BMI**					
OSA	Non-Obese	5 (62,5%)	3 (37.5%)	8 (100%)	0.088
	Obese	6 (25%)	18 (75%)	24 (100%)	
	Total	11 (34.4%)	21 (65.6)	32 (100%)	
Non-OSA SDB	Non-Obese	2 (28.6%)	5 (71.4%)	7 (100%)	1.000
	Obese	2 (33.3%)	4 (66.7%)	6 (100%)	
	Total	4 (30.8%)	9 (69.2%)	13 (100%)	
Total	Non-Obese	7 (46.7%)	8 (53.3%)	15 (100%)	0.200
	Obese	8 (26.7%)	22 (73.3%)	30 (100%)	
	Total	15 (33.3%)	30 (66.7%)	45 (100%)	
** Age**					
OSA	Age < 55	7 (36.8%)	12 (63.2%)	19 (100%)	0.513
	Age > 55	4 (30.8%)	9 (69.2%)	13 (100%)	
	Total	11 (34.4%)	21 (65.6%)	32 (100%)	
Non-OSA SDB	Age < 55	3 (33.3%)	6 (66.7%)	9 (100%)	0.646
	Age > 55	1 (25%)	3 (75%)	4 (100%)	
	Total	4 (30.8%)	9 (69.2%)	13 (100%)	
Total	Age < 55	10 (35.7%)	18 (64.3%)	28 (100%)	0.461
	Age > 55	5 (29.4%)	12 (70.6%)	17 (100%)	
	Total	15 (33.3%)	30 (66.7%)	45 (100%)	

* Fisher test exact sig 2-sided

Table 2 demonstrates that the BMI of patients with and without LPR was similar. In this study, 75% of OSA patients were obese; in the LPR group, 25% of OSA patients were obese. SDB patients with high BMI tended to have LPR and/or OSA. No significant association between BMI and OSA (p=0.088), BMI and non-OSA SDB (p=1.000), respectively.

Table 1 shows that the patient age range in the four groups was similar. In patients with LPR, most patients who also had non-OSA SDB had a median age of 48 years; patients with OSA had a median age of 52 years. In Table 2, a significant association between age and LPR in both non-OSA SDB and OSA patients was not reached (p=0.513 and p=0.646, respectively).

Both ESS and STOP-BANG scores were similar among the two groups Table 1. The average ESS scores suggested EDS in both groups. The LPR group had higher ESS scores than the non-LPR group; however, this difference was not statistically significant (p=0.064). Furthermore, there was no significant difference in STOP-BANG scores between the LPR and non-LPR groups. 

Regardless of non-LPR or LPR status, SDB patients (non-OSA or OSA) had a low percentage of REM sleep: 2.40%, 3.10%, 5.90%, and 5.20%, respectively Table 1. In contrast, the percentage of light sleep increased in these groups. In the LPR group, patients with non-OSA SDB and OSA had a higher percentage of light sleep than patients in the non-LPR group. For percentage of deep sleep, the non-OSA SDB group exceeded the normal range; in contrast, the percentage of deep sleep in OSA patients was within the normal range.

## Discussion

LPR incidence among OSA patients from different studies ranges from 32.9% to 89.2%, which is higher than in the general population.(6) In this study, the incidence of LPR among OSA patients was 34.4%, which is consistent with the results of previous studies. Variability of incidence between these studies is high and is likely influenced by differences in study population size, diagnostic methods, and age range. A study by Kim et al. (16) used RSI only, Caparroz et al. (9) and Rizki et al. (17) used RSI and Reflux Finding Score (RFS), while Cumpston et al. (18) used multichannel intraluminal impedance (MII) to diagnose LPR. The population sizes also varied from 46–109 subjects in different age ranges.

Theories describing the relationship between LPR and OSA vary among studies. Eskiizmir et al.(12) stated that there is a vicious cycle between OSA and LPR; recurrent LPR causes mucosal injury due to inflammation, and chronic inflammation causes direct tissue edema and airway narrowing (3,12). It is thought that chemical irritation generates sensory deficits in upper airway mucosa and disrupts reflexes that are necessary to maintain upper airway patency (19). A study by Horner et al.(20) demonstrated the mechanoreceptor reflex, where negative intraluminal pressure produced activation of the pharyngeal dilator muscle (genioglossus as the most dominant muscle) via the vagal and trigeminal nerves. Chronic inflammation due to LPR disrupts this afferent reflex by causing dysfunction in the sensing of negative intraluminal pressure, thereby increasing upper airway collapsibility, which is the primary pathophysiology of OSA (17,21). However, this theory was not supported by Magliulo et al. (6), who stated that there was no significant correlation between the severity of AHI in OSA patients with LPR. In our study, almost all parameters of OSA within the two groups were similar. 

Regarding gender, the occurrence of SDB with LPR was higher in males than in females. LPR in males, both in the non-OSA SDB group (38%) and the OSA group (46%), was twice as common as in females (20% and 24%, respectively), despite not reaching statistical significance. Appleton et al. (22) reported that males were more likely to be obese than females and were thus at a higher risk of developing both LPR and OSA. Other studies did not support this finding as they found higher OSA and LPR in females than males through laryngology clinical evaluation followed by MII (21). The lower incidence of OSA in females might be due to fewer reports of common OSA symptoms, such as snoring or witnessed apneas, as they have more atypical OSA complaints, such as daytime fatigue, insomnia, mood disturbances, and nightmares, which may be also influenced by estrogen and progesterone (23).

Our study found that 75% of patients with OSA were obese. Obesity was also observed in 55% of the LPR patients with OSA. This observation indicates that SDB patients with high BMI tend to have LPR and/or OSA; meanwhile, patients with high BMI have an increased risk of developing LPR. Most studies are in agreement regarding the correlation between obesity and LPR (24,25). However, BMI is considered a confounding variable that can affect the association between LPR and OSA (24). Rodrigues et al.(25) reported the RSI of obese patients was significantly higher in patients with moderate to severe OSA, however the same correlation was not observed in the evaluation of RFS. Age is thought to be a risk factor for LPR in OSA. However, our study did not find any statistical significance between age and non-OSA SDB or OSA. A study by Bixler et al. (26) reported an increasing risk of OSA until the age of 55 years and a reduction thereafter due to the increase in awareness to seek medical advice for OSA in people under 55 years of age.

In our study, questionnaires, such as the ESS and STOP-BANG, were applied to evaluate SDB. ESS evaluates EDS, and the average ESS scores were similar between groups, suggesting EDS. The ESS is a non-specific test used to measure EDS in SDB, as other factors, such as chronic disease, stressful life, low physical activity, and non-SDB sleep deprivation can also lead to EDS (27). There was no association between RSI and ESS or RSI and STOP-BANG. However, following analysis via an independent non-parametric test, patients with LPR tended to have higher ESS scores. Steward et al. (28) treated 27 SDB patients with a proton pump inhibitor and found a reduction in EDS evaluated via ESS and reflux symptoms score. Future studies should consider increasing the sample size, as this relationship may reach statistical significance. In contrast, STOP-BANG results were not associated with RSI scores. This is consistent with a study by Laohasiriwong et al. (29), which revealed no correlation between RSI and STOP-BANG scores. 

Our study indicated that SDB patients had a lower percentage of REM sleep regardless of their LPR status, and the lowest percentage was in the non-OSA SDB group. Several studies on sleep and OSA have been conducted in recent years. A study by Zhang et al. (30) in rats found that chronic REM sleep deprivation induces LPR due to dysmotility of the gastrointestinal tract controlled by the autonomic nervous system. There are two mechanisms that explain this: (1) an impaired sympathetic cardiovascular system, inducing the secretion of catecholamines, leading to elevated blood pressure in all sleep stages; and (2) bradyarrhythmia occurring after obstructive events, which triggers intrinsic conduction system abnormalities resulting in ventricular and atrial arrhythmias. Fagioli et al. (31) demonstrated that oxygen consumption during sleep was highest in REM sleep, followed by N2 sleep and deep sleep. Therefore, as SDB patients have lower blood oxygen saturation, this might cause inadequate REM sleep. Lower percentage REM sleep can also be induced by decreased genioglossal muscle tone and nasal obstruction, resulting in obstruction and hypoxia, which shortens and fragments REM sleep (32).

In contrast with the decrease in REM sleep percentage, light sleep (N1, N2) percentage increased in our study, and 65% of patients in the non-LPR group had longer deep sleep (N3). Increasing light sleep was due to a decline in blood oxygen saturation (SpO_2_) responsible for brain arousal, which shifted REM sleep percentage to favor light sleep (33). 

Shahveisi et al. (33) demonstrated that, by controlling age and BMI, the percentage of N1 sleep in the OSA group was significantly higher than the normal and primary snoring group. The increased percentage of deep sleep might be caused by increasing GABA, which inhibits the esophago-upper esophageal sphincter relaxation reflex and increases LES contraction, decreasing the risk of LPR (34,35). In contrast, in the LPR group, 33% of patients had decreased deep sleep percentage. This might have been influenced by a low amount of GABA, which was inhibited by catecholamines secreted due to autonomic dysfunction during apnea episodes, causing hypertension and arousals. Wu et al.(36) suggested that increased N3 sleep percentage indicates lower severity of OSA, as fewer respiratory events occur in the N3 sleep stage.

Overall, almost all OSA parameters in the non-LPR and LPR groups in this study were similar. These results might be caused by (1) information bias, subjectivity of patients, and the influence of the patients' anxiety or fear. To normalize this variable, the study should evaluate the psychological aspects of each patient in the study using the Hospital Anxiety Depression Scale (HADS). (2) The commencement of anti-reflux therapy may have influenced the RSI. (24). (3) Unequal diagnostic modalities may have influences the results, as RSI is a questionnaire-based diagnostic tool, while PSG is the gold standard method for diagnosing SDB. Furthermore, (4) RSI measures the symptoms and effects of reflux in the larynx or pharynx, not the reflux itself; in contrast, PSG is a time-point examination, evaluating the episode when apnea occurs. 

The main limitations of the present study were: (1) there was incomplete RFS data, and complete RFS data would have further supported the LPR diagnosis; and (2) we had no access to the gold standard examination for GERD or LPR, i.e. Multichannel Intraluminal Impedance (MII). Even though comparing RSI to PSG is possible, it will not provide any significant conclusions as they are not equal modalities. 

## Conclusion

The incidence of LPR in OSA patients and non-OSA SDB patients is similar. Sleep architecture in patients with SDB shows decreased REM sleep percentage, increased deep sleep, and increased light sleep regardless of LPR status. 

Occurrence of LPR in SDB patients by gender. LPR is more likely to occur in males with SDB than in females. In the non-OSA SDB group, the prevalence of LPR in males (38%) is almost twice the prevalence in females (20%). In the OSA group, 7/15 (46%) males with OSA had LPR; the prevalence of LPR in females with OSA was 24%.
